# Micro-replication platform for studying the structural effect of seed surfaces on wetting properties

**DOI:** 10.1038/s41598-022-09634-7

**Published:** 2022-04-04

**Authors:** Seungwoo Shin, Su Hyun Choi, Shukherdorj Baasanmunkh, Seok Kim, Hyeok Jae Choi, Young Tae Cho

**Affiliations:** 1 Department of Smart Manufacturing Engineering, Changwon National University, Changwon-si, 51140 Republic of Korea; 2 Department of Mechanical Engineering, Changwon National University, Changwon-si, 51140 Republic of Korea; 3 Department of Biology and Chemistry, Changwon National University, Changwon-si, 51140 Republic of Korea

**Keywords:** Nanobiotechnology, Nanostructures, Soft lithography

## Abstract

Biological surfaces in plants are critical for controlling essential functions such as wettability, adhesion, and light management, which are linked to their adaptation, survival, and reproduction. Biomimetically patterned surfaces replicating the microstructures of plant surfaces have become an emerging tool for understanding plant–environment interactions. In this study, we developed a two-step micro-replication platform to mimic the microstructure of seed surfaces and demonstrated that this initial platform can be used to study seed surface–environment interactions. The two-step process involved the extraction of a simplified seed surface model from real seeds and micro-replication of the simplified seed surface model using nanoimprint lithography. Using *Allium* seeds collected from Mongolia and Central Asia as the model system, we studied the wettability of biological and synthetic seed surfaces. We could independently control the material properties of a synthetic seed surface while maintaining the microstructures and, thereby, provide clear evidence that *Allium* seed surfaces were highly wettable owing to the high surface energy in the epidermal material rather than a microstructural effect. We expect that this platform can facilitate study of the independent effect of microstructure on the interaction of seed surfaces with their surroundings and contribute to research on the evolution of plant–environment interactions.

## Introduction

Biological surface structures of plants influence the physical or optical behavior of multifunctional interfaces between organisms and their surroundings, such as wettability, particle adhesion, and light reflection^1,2^. In particular, surface wetting is critical for many biological processes in plants, including seed germination, pollinator attraction, water transport control, and water and nutrients uptake in soils^1^. There are four types of structures and wetting behaviors of plant surfaces: hydrophilic, hydrophobic, superhydrophobic, and super-hydrophilic. These are characterized by the contact angle (CA) of a water droplet on a solid surface and measured as static or dynamic^3–5^. Surfaces, particularly those with a low CA (< 90°) are hydrophilic, those with a high CA (> 90°) are hydrophobic, and surfaces with a CA over 150° are superhydrophilic^3^; the latter are observed in aquatic plants and many land plants^4^. In the past, many studies have been conducted on plant surfaces and surface wettability, mostly based on flowers, leaves, and other organs^3,6,7^. The wetting properties of plant cuticles depend on structural and chemical modifications integrated with the exposed waxes^1^. Although the chemical nature of most plant surfaces is hydrophobic because of the waxy materials used as a barrier against water loss, the presence of micro and nanostructures of the surfaces (e.g., folding of the cuticle) have a substantial influence on surface wet-tability^8,9^.

The first point of contact in the interaction between the plant and its environment is the epidermal surface of the plants. This consists of physical interactions due to the surface structure and chemical/molecular interactions linked with molecular signals expressed on the surface. However, when studying the interaction of the plant surface with the environment, it is difficult to separate these two factors because they are strongly entangled in biological systems. An example is found in plant seeds. Seeds are one of the most vital components of a plant, as they not only initiate the life cycle and reproduction of a species but also facilitate dispersal and persistence in new environments^10^. Among the various physiological characteristics of plant seeds, the micro-morphologies of the seed surfaces (i.e., testa cells) are concave patterns with a complete or particular deflection of the outer epidermis wall, which might originate from cell shrinking induced by water loss^11^. Along with the surrounding environment, seed surface characteristics such as morphology, structure, and composition also influence the wetting behavior and kinetics of water uptake by the seeds during germination^12^. Although seed surface structures have recently been studied with a detailed classification of the macro-and micro-morphology^13^, the physical interaction of seed microstructures with their environment has been rarely understood. This happens because no appropriate methodology exists for studying seed surfaces and isolating their structural effects in complex seed–environment interactions.

Biomimetic replicated surface is a powerful tool for understanding plant–environment interactions^14,15^. For example, replicating the hierarchical microstructures of lotus leaves have enabled the fabrication of superhydrophobic surfaces for practical applications, including anticorrosive, antifogging, antifouling, and oil/water separation purposes^16–18^. These artificial biological surfaces have recently expanded the ability to create a synthetic platform for mimicking biological structures or studying how microstructures influence microorganism–plant interactions^14,15,19,20^.

**Figure 1:**
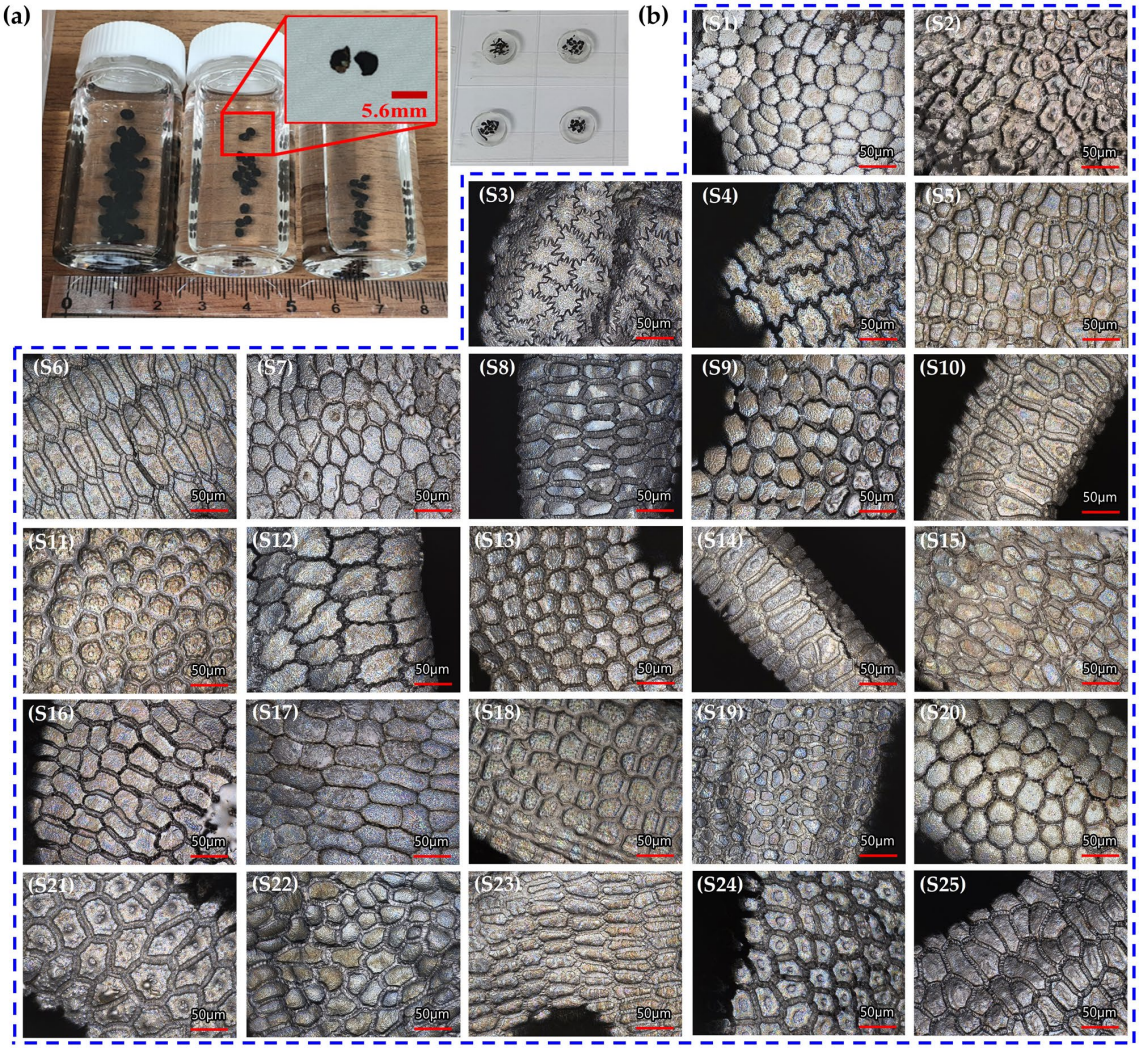
Microscope images of *Allium* seeds; (**a**) Image of *Allium* seed specimens and (**b**) microscope images of *Allium* seeds samples 1–25.

In this study, the replication of the seed surface microstructures was first demonstrated using a two-step process involving the simplification of the seed surface patterns and their replication by nanoimprint lithography (NIL)^21–24^. We used *Allium* L. seeds as our model system, collected from Mongolia and Central Asian countries, such as Kyrgyzstan and Uzbekistan, which are well known for their dry and mostly desert environments. Their small size and high curvatures, as shown in Fig. 1a, makes it challenging to directly obtain templates (negatives) of seed surfaces by harnessing a conventional method such as that used for the replication of flat and large leaves. Therefore, we first analyzed the microscopic three-dimensional (3D) morphologies of 25 seed samples (Fig. 1) and their corresponding wettability and then extracted the two characteristic surface models for the replication process. Through the conventional silicon Si-based microfabrication process, we transferred our simplified seed surface models to a hard Si master mold as negative templates. Then, we obtained positive replication using NIL with ultraviolet (UV) curable polyurethane acrylate (PUA) resin, with the seed surface microstructures in a synthetic material.

To demonstrate the feasibility of our method as a tool to study seed surface–environment interactions, we conducted a comparative study on the wettability of bio-logical and artificial seed surfaces. As water and nutrients are important for seed germination and growth, seed surfaces should control the ability to absorb or block water depending on the specific surroundings. We speculated that the seed surface may be strongly correlated with wetting properties^25^. To separate the structural effect on the wetting properties from chemical/molecular interactions, we also studied the wetting behavior after adjusting the surface energy of the replicated seed surfaces through oxygen plasma treatment^26–29^. Furthermore, to verify the effect of the constituent material on the wetting of seed surfaces, we investigated particle aggregation patterns on the biological seeds and replicated surfaces after evaporation of sprayed nanoparticle-laden droplets^30^. These results support our understanding of the interplay between surface characteristics and wetting properties and provide direct evidence related to the wettability of the outermost waxes or constituent materials of the seed surfaces.

## Results and discussion

**Figure 2:**
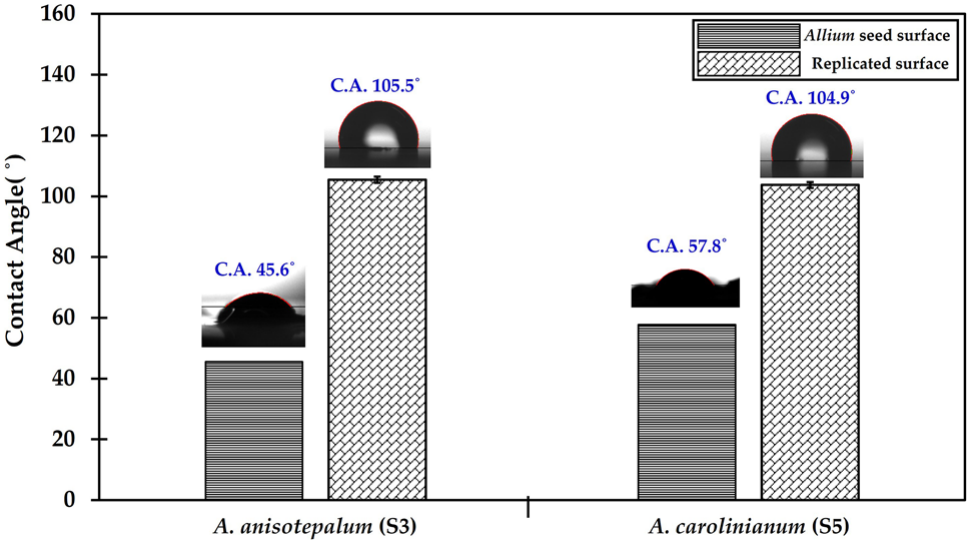
The measured contact angle on original and synthetic surfaces of *Allium* seeds.

We first compared the wetting properties of artificial seed surfaces with those of real seeds in terms of potential to study seed surface–environment interactions. The wettability of the original and synthetic seed surfaces was evaluated by CA measurements on a static sessile deionized (DI) water drop, as shown in Fig. 2. The replicated PUA seed surfaces were hydrophobic to DI water with a CA of approximately 105°. Com-pared to these synthetic seed surfaces, *Allium* seed surfaces showed much greater wettability for water drops (Table 1; Fig. 2) probably because of the epidermal materials. The considerable difference in wettability between biological and synthetic seed surfaces implies the importance of the constituent material effect. Generally, the wetting behavior of surfaces is determined by the interplay with topological structures (e.g., roughness, surface texture, fraction of solid area) and their constituent material proper-ties (e.g., surface tension of the liquid, surface energy of the solid surface)^31^. The CA measurement allows us to understand the relationship between the liquid drop and the surface and to calculate the surface free energy by evaluating the interface of a liquid and a solid surface. If the surface energy of the solid surface is strong, this high energy pulls hard on the water drop, causing it to spread out further over the surface; this surface is referred to as wettable or hydrophilic. If the surface energy of the solid surface is not stronger than the surface tension of the liquid, the drop constricts into a shape similar to a sphere; this surface is referred to as a non-wettable or hydrophobic surface. The resulting low CA indicates that the constituent material of the real seed surfaces has a higher surface energy compared to that of the synthetic seed surfaces.

**Table 1:** Contact angles and shapes of *Allium* seeds.

Seed	Scientific name	Shape	Contact angle (°)
Sample 1 (S1)	*A*. *anisopodium* Ledeb	Polygon	41.93 ± 13.80
Sample 2 (S2)	*A*. *barsczewskii* Lipsky	Polygon	Hydrophilic
Sample 3 (S3)	*A*. *anisotepalum* Vved	Zigzag	45.55 ± 6.43
Sample 4 (S4)	*A*. *caesium* Schrenk	Zigzag	Hydrophilic
Sample 5 (S5)	*A*. *carolinianum* DC	Polygon	57.75 ± 2.33
Sample 6 (S6)	*A*. *clathratum* Ledeb	Polygon	Hydrophilic
Sample 7 (S7)	*A*. *bidentatum* Fisch. ex Prokh	Polygon	Hydrophilic
Sample 8 (S8)	*A*. *flavovirens* Regel	Polygon	Hydrophilic
Sample 9 (S9)	*A*. *galanthum* Kar. & Kir	Polygon	Hydrophilic
Sample 10 (S10)	*A*. *amphibolum* Ledeb	Polygon	38.60 ± 2.40
Sample 11 (S11)	*A*. *oschaninii* O.Fedtsch	Polygon	Hydrophilic
Sample 12 (S12)	*A*. *pallasii* Murray	Polygon	Hydrophilic
Sample 13 (S13)	*A*. *korolkowii* Regel	Polygon	47.50 ± 6.79
Sample 14 (S14)	*A*. *malyschevii* N.Friesen	Polygon	42.50 ± 4.81
Sample 15 (S15)	*A*. *obliquum* L	Polygon	47.10 ± 0.99
Sample 16 (S16)	*A*. *tenuissimum* L	Polygon	52.30 ± 5.52
Sample 17 (S17)	*A*. *vodopjanovae* N.Friesen	Polygon	22.80 ± 1.84
Sample 18 (S18)	*A*. *petraeum* Kar. & Kir	Polygon	Hydrophilic
Sample 19 (S19)	*A*. *platyspathum* Schrenk	Polygon	Hydrophilic
Sample 20 (S20)	*A*. *polyrhizum* Turcz. ex Regel	Polygon	33.45 ± 0.35
Sample 21 (S21)	*A*. *tracyscordum* Vved	Polygon	47.08 ± 9.21
Sample 22 (S22)	*A*. *altaicum* Pall	Polygon	41.25 ± 16.60
Sample 23 (S23)	*A*. *tianschanicum* Rupr	Polygon	Hydrophilic
Sample 24 (S24)	*A*. *dolichostylum* Vved	Polygon	Hydrophilic
Sample 25 (S25)	*A*. *strictum* Schrad	Polygon	55.83 ± 11.51

**Figure 3:**
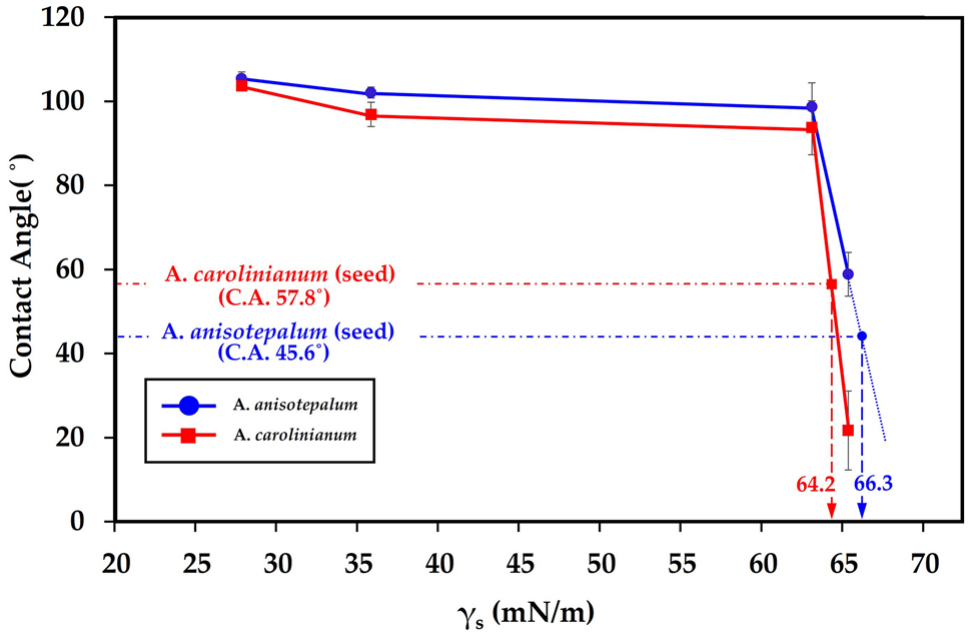
Apparent contact angle according to the surface energy of synthetic *Allium* seed surfaces.

Therefore, we investigated the wetting behavior of the replicated synthetic seed surfaces by varying the surface energy of the constituent material while maintaining identical microstructures. In this study, we used an ultraviolet ozone (UVO) treatment system (UVC-30, Jaesung Engineering Co., Republic of Korea) to adjust the surface energy of the PUA resin. UVO surface treatment is an effective method for increasing the hydrophilicity (i.e., increasing the surface energy of the solid surface) of polymeric materials without affecting the surface roughness and structures^32^. Here, the surface energy of the PUA material at different UVO treatment times was calculated by measuring the advancing CA on planar films using the needle-in-drop method^33^. Figure 3 shows the behavior of the apparent CAs of the synthetic seed surfaces as a function of surface energy. As the surface energy increased, the apparent CA of the synthetic seed surfaces gradually decreased and became similar to that of the real seed surfaces (Fig. 3). By comparing the CA as function of surface energy of the synthetic seed surfaces with the CA of the biological pattern, the surface energies of A. carolinianum and A. anisotepalum are expected to be about 64.2, 66.3 mN/m. These results strongly indicate that the outermost epidermal composition of *Allium* seed surfaces may be comprising high surface energy materials (i.e., highly wettable).

**Figure 4:**
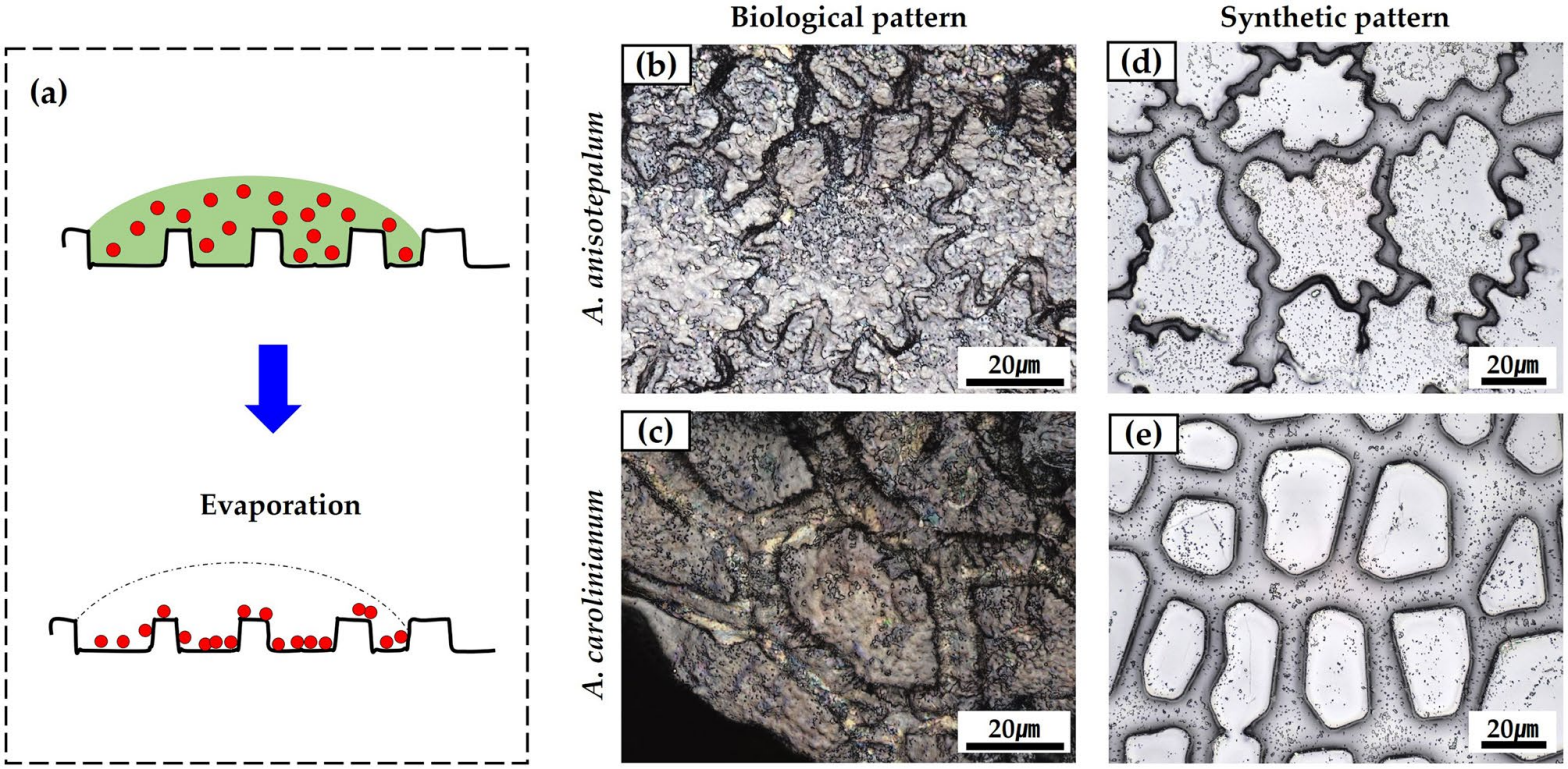
(**a**) Schematics for particle residues on hydrophilic surfaces. Particle aggregation patterns on (**b**, **c**) biological and (**d**, **e**) synthetic seed surfaces after drying sprayed droplets of particle-laden solution.

Further, to provide clearer evidence of the high surface energy of the epidermal seed surfaces, we investigated the aggregated particle patterns after drying sprayed droplets of a particle-laden solution on the biological and synthetic seed surfaces. According to our previous study^30^, the evaporation-induced particle residues differed depending on the surface wettability. For hydrophilic or super-hydrophilic surfaces, the sprayed microdroplets tend to coalesce, leading to the typical Wenzel state on the structured sur-faces (Fig. 4a). After the evaporation of the microdroplets, the nanoparticles were uniformly deposited and aggregated along the surface topology owing to the pinning effect of the contact line. On the hydrophobic surfaces, the sprayed droplets remained small microdroplets and tended to be trapped within the microstructures. After the evaporation of the microdroplets, most of the particles were deposited and aggregated at the bottom of the microstructures because of the depinning of the evaporating droplets (Fig. S2b). We observed that the nanoparticle aggregates were uniformly deposited on both the real (Fig. 4b, c) and synthetic seed surfaces (Fig. 4d, e). This also confirms that the epidermal constituent material of *Allium* seeds is hydrophilic (i.e., a high surface energy material).

Hydrophilic or super-hydrophilic surfaces, caused by different micro and nanostructures, have been developed and evolved in different organs of plants for adaptation for survival in their surroundings^34^. For example, carnivorous pitcher plants within the genus *Nepenthes* L. use super-wettable surfaces to rapidly spread water to a flat film for insect capture. On the leaves of *Ruellia devosiana*, the spreading of water by the super-hydrophilic leaf surface provides faster evaporation of water due to an increase in the water–air interface. Based on the survival strategies of other plants and their parts, we speculate that the micro-structure of *Allium* seed surfaces may be developed as an efficient mechanism for self-survival against their surroundings via the control of wettability. In this study, *Allium* seeds were collected from Mongolia and Central Asia, which are in the center of the continent and are the areas furthest from the sea in the world. They are, therefore, mostly occupied by desert and desert steppe areas, which are water-limited ecosystems^35,36^. Seeds need ideal conditions to germinate, and for most, that means sufficient water. For desert plants, sufficient water can take months or years to arrive, so some seeds become dormant^37^. Based on our results, we postulate that plant seeds have developed various mechanisms to detect moisture conditions via the epidermis of their surfaces if they need to absorb water for germination.

Forthcoming studies including samples with more various patterns are required to accurately infer the independent effect of microstructure on the interaction of seed surfaces with their surroundings and contribute to research on the evolution of plant–environment interactions.

## Conclusion

In this study, we developed a two-step micro-replication platform, enabling the fabrication of synthetic surfaces to study biological surface–environment interactions. As a first concept demonstration, we performed a comparative study of the wettability of real and synthetic seed surfaces. *Allium* seeds collected from Mongolia, Kyrgyzstan, and Uzbekistan were used as model systems. We first analyzed the 3D morphologies and wettability of 25 seed samples and extracted two characteristic seed surface models for simplification. Based on a simplified seed surface model, Si master molds were created as negative templates via a conventional microfabrication process. Then, the polymeric synthetic seed surfaces were replicated by NIL with UV-curable PUA resins. By systematically comparing the real and replicated seed surfaces, we speculate that the high wettability of *Allium* seed surfaces is attributed to the effect of the high surface energy of the outermost epidermal materials rather than the microstructural effect.

Thus, we believe that the micro-replication of seed surfaces with the synthetic material provides (1) the capability to understand how surface topological features of real seed surface materials affect wettability and (2) independent control of material properties of seed surfaces, while maintaining identical microstructures in the replicas. In addition, this platform can be widely available because it does not require expensive machinery or specialized tools and can be used in any laboratory. We expect that this research can provide a novel platform to addressing research questions on the interaction between plant organs and their environment, specifically biological surface–environment interactions. This allows researchers to study the structural and material effects in the interaction, discovering uncovered functions or mechanisms of this interaction.

## Methods

### Analysis of the surface microstructure and wettability

The surface structure of *Allium* seeds was analyzed using a laser scanning confocal microscope (Keyence VK-X1000), and the CA was measured to analyze the seed surface wettability. Table 1 summarizes the scientific names, shapes, and CAs of 25 *Allium* seeds with different scientific names. Seeds were collected from Mongolia and Central Asia, such as Kyrgyzstan and Uzbekistan^11^. These regions mostly comprise desert and desert steppe areas^35,36^. For this reason, we speculated that the seeds grown in dry conditions might have highly wettable surfaces for sufficient water collection and absorption to survive, which was confirmed by the measured results which showed that most seed samples exhibited a wettable surface (Table 1).

First, it is important to carefully observe the microstructure of the seed surface to mimic it. Figure 1 shows the detailed 3D microstructures of 25 *Allium* seeds using a laser scanning confocal microscope. The measured *Allium* seed topography usually consists of undulated micro-patterns and reliefs of the cell walls with different amplitudes and wavelengths, which range from ~ 20 to ~ 100 μm depending on the species. To simplify the morphological characteristics, we categorized the undulations of *Allium* seed surfaces with only two types of shapes: polygons (S1) and zigzags (e.g., S3). As depicted in the measured results in Table 1, there was no significant tendency of contact angle according to the shapes of the undulations, which might be due to the combined effect of the structure and the constituent material of the seed surfaces. Hence, we studied the wetting behavior using replicated synthetic seed surfaces to isolate the structural effect of the epidermal material on the wetting properties.

### Fabrication of artificial seed surface

**Figure 5:**
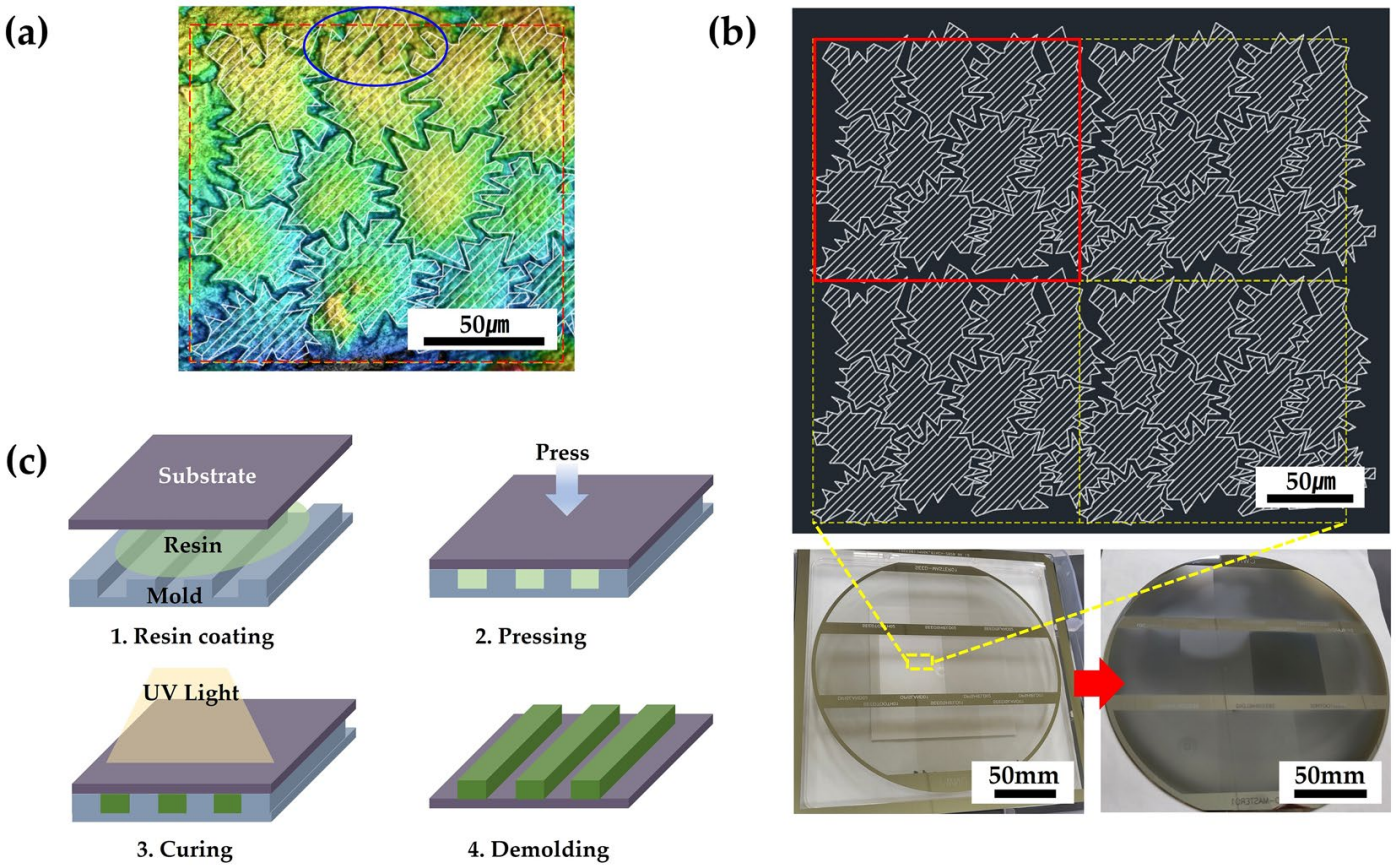
The two-step replication process of *Allium* seed surfaces. (**a**) Microscopic image of measured *Allium* seed surface; (**b**) first step entails the preparation of Si master mold designed by simplified modeling of seed sur-faces extracted from the measured image and (**c**) second step involves the replication of the polymeric seed surfaces from the Si master mold using the NIL process.

To replicate the micro-patterns of the seed surfaces with robust polymeric materials while excluding the influence of the nonuniform surface material from seed to seed, we selected two seed samples of *Allium anisotepalum* (S3) and *Allium carolinianum* (S5) as representing the characteristic *Allium* seed microstructures in this study. Then, the synthetic seed surfaces were fabricated by a two-step process of simplification of the seed surface patterns and replication of the simplified seed surface models using NIL. We extracted a unit cell of the characteristic microstructures of seed surfaces based on the microscopic images of biological seed samples (Fig. 5a). The photomask for the photolithography process consisted of arrays of extracted seed surface patterns with areas of 200 mm × 150 mm (bottom left in Fig. 5b). Si master molds for the templates (bottom right in Fig. 5b) were fabricated using conventional photolithography^38–41^ and subsequent reactive ion etching (RIE)^42–45^. Briefly, the resist-coated Si wafer was patterned using a photomask of modeled seed surfaces, and the patterned wafer with a photoresist was physically etched using the RIE process. The artificial seed surfaces were fabricated using the NIL process with PUA (MINS-311RM, Minuta Technology, Korea) resin (Fig. 5c). The PUA resin was dispensed dropwise onto a polyethylene terephthalate film, and the Si hard mold was pressed against the liquid drops. The PUA resin was cured with ultraviolet (UV) light (λ = 365 nm) for few seconds. The detailed UV NIL process has been described in our previous work^30,46^.

**Figure 6:**
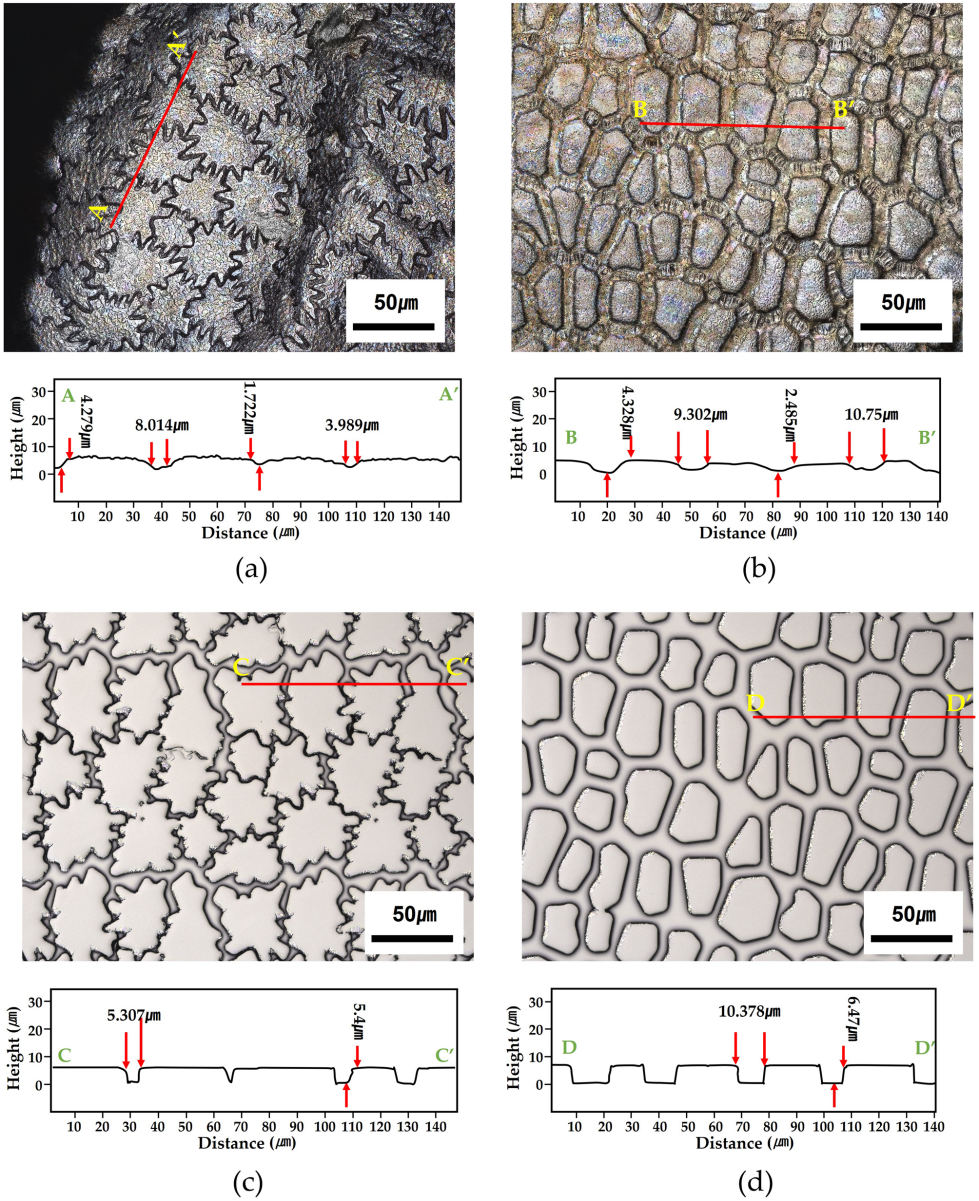
3D surface profiles of biological and synthetic seed samples; Microscope image and cross-sectional profile of (**a**) *Allium anisotepalum* and (**b**) *A. carolinianum* seeds; microscope image and cross-sectional profile of the seed surface replicas of (**c**) *A. anisotepalum* and (**d**) *A. carolinianum.*

Figure 6 shows the microscopic images and 3D profiles of the original (Fig. 6a, b) and corresponding synthetic surfaces (Fig. 6c, d) of *Allium* seeds. The topological features of the replicas, such as the shape, width, and depth of the undulation, are consistent with those of the real seed surfaces, suggesting that the surface microstructures of the replicas were faithfully reproduced via micro-replication. All *Allium* seeds (Table S1) used in the experiment performed in accordance with the relevant guidelines and regulations.

## Supplementary Information

Supplementary Information.
