# Supplementary material for: Micro-replication platform for studying the structural effect of seed surfaces on wetting properties
Source: Sci Rep. 2022 Apr 4;12:5607. doi: 10.1038/s41598-022-09634-7 (PMC8980016; doi:10.1038/s41598-022-09634-7)
Supplement: Supplementary file 1 — Supplementary Information. [file 41598_2022_9634_MOESM1_ESM.docx]

***Supplementary Materials***

**Micro-replication platform for studying the structural effect of seed surfaces on wetting properties**

Seungwoo Shin^1^, Su Hyun Choi^2^, Shukherdorj Baasanmunkh^3^, Seok Kim^1,2^, Hyeok Jae Choi^3^*, Young Tae Cho^1,2^*

^1^ Department of Smart Manufacturing Engineering, Changwon National University, 51140, Republic of Korea

^2^ Department of Mechanical Engineering, Changwon National University, 51140, Republic of Korea

^3^ Department of Biology and Chemistry, Changwon National University, 51140, Republic of Korea

* Corresponding authors: Hyeok Jae Choi(hjchoi1975@changwon.ac.kr); Young Tae Cho (ytcho@changwon.ac.kr).

**Table S1.** Information of *Allium* species investigated (CWNU: Changwon National University).

| **Subgenus/Section** | **Scientific name** | **Collection site** | |
| --- | --- | --- | --- |
| *Rhizirideum*/*Tenuissima* | *A. anisopodium* Ledeb. | | Mongolia: Khovd province, Mankhan sum, 3 tsenkheriin agui, 31 Jul 2016, *H.J. Choi et al.* (CWNU) |
| *Reticulatobulbosa*/*Campanulata* | *A. barsczewskii* Lipsky | | Uzbekistan: Yangikurgan region Pozamon Chatkal range, 08 August 2014, *H.J. Choi et al.* (CWNU) |
| *Allium*/*Minuta* | *A. anisotepalum* Vved. | | Uzbekistan: Namangan city, Yangikurgan, Hill of Ungoe, Tashkent Botanical Garden, 24 May 2015*, H.J. Choi et al.* (CWNU) |
| *Allium*/*Caerulea* | *A. caesium* Schrenk | | Uzbekistan: Tashkent Botanical Garden, 26 May 2015, *H.J. Choi et al.* (CWNU) |
| *Polyprason*/*Falcatifolia* | *A. carolinianum* DC. | | Kyrgyzstan: Kara-balta River, 01 Aug 2017, *H.J. Choi et al.* (CWNU) |
| *Reticulatobulbosa*/*Reticulatobulbosa* | *A. clathratum* Ledeb. | | Mongolia: Khovd province, Munkhkhairkhan, Senkheriin khavtsal, 29 Jul 2016, *Sh. Baasanmunkh et al.* (CWNU) |
| *Rhizirideum*/*Caespitosoprason* | *A. bidentatum* Fisch. ex Prokh. | | Mongolia: Tuv province, Hustai National Park, 11 Aug 2016, *H.J. Choi et al.* (CWNU) |
| *Reticulatobulbosa*/*Reticulatobulbosa* | *A. leucocephalum* Turcz. ex Vved. | | Mongolia: Tuv province, Hustai National Park, 11 Aug 2016, *H.J. Choi et al.* (CWNU) |
| *Cepa*/*Cepa* | *A. galanthum* Kar. & Kir. | | Kyrgyzstan: 2 km west from Krasnyy Village, 03 Aug 2017, *H.J. Choi et al.* (CWNU) |
| *Reticulatobulbosa*/*Reticulatobulbosa* | *A. amphibolum* Ledeb. | | Mongolia: Khovd province, Munkhkhairkhan, Senkheriin khavtsal, 25 Jul 2016, *Sh. Baasanmunkh et al.* (CWNU) |
| *Cepa*/*Cepa* | *A. oschaninii* O.Fedtsch. | | Uzbekistan: Tashkent Botanical Garden, 05 Jul 2015, *H.J. Choi et al.* (CWNU) |
| *Allium*/*Pallasia* | *A. pallasii* Murray | | Kyrgyzstan: Kara-balta River, 01 Aug 2017, *H.J. Choi et al.* (CWNU) |
| *Polyprason*/*Falcatifolia* | *A. korolkowii* Regel | | Uzbekistan: Cholpon Ata, 10 Jul 2016, *H.J. Choi et al.* (CWNU) |
| *Reticulatobulbosa*/*Reticulatobulbosa* | *A. malyschevii* N.Friesen | | Mongolia: Khuvsgul province, Khatgal sum, 15, July 2015, *H.J. Choi et al.* (CWNU) |
| *Polyprason*/*Oreiprason* | *A. obliquum* L. | | Kyrgyzstan: The Narin mountain, north makrosclone, Narin NES, 10 Aug 2009, *G.A. Lazkov* (CWNU) |
| *Rhizirideum*/*Tenuissima* | *A. tenuissimum* L. | | Mongolia: Khentii province, 25 km from Berkh sum, 11 Jul 2017, *H.J. Choi et al.* (CWNU) |
| *Rhizirideum*/*Tenuissima* | *A. vodopjanovae* N.Friesen | | Mongolia: Khovd province, Munkhkhairkhan, Tsagaan sair, 30 Jul 2016, *H.J. Choi et al.* (CWNU) |
| *Polyprason*/*Oreiprason* | *A. petraeum* Kar. & Kir. | | Kyrgyzstan: Gornolyzhnyy Kurort "Politekh" 02 Aug 2017, *H.J. Choi et al.* (CWNU) |
| *Polyprason*/*Falcatifolia* | *A. platyspathum* Schrenk. | | Kyrgyzstan: 13 km south side from Jergalan, 30 Jun 2018, *H.J. Choi et al.* (CWNU) |
| *Rhizirideum*/*Caespitosoprason* | *A. polyrhizum* Turcz. ex Regel | | Mongolia: Govisumber province, Shivee-Ovoo, 21 Aug 2016, *Sh. Baasanmunkh et al.* (CWNU) |
| *Reticulatobulbosa*/*Scabriscapa* | *A. trachyscordum* Vved. | | Kyrgyzstan: 35 km southeast side from Novovoznesenovka, 30 June 2018, *H.J. Choi et al.* (CWNU) |
| *Cepa*/*Cepa* | *A. altaicum* Pall. | | Mongolia: Ulaanbaatar, 11 Apr 2017, *H.J. Choi et al.* (CWNU) |
| *Polyprason*/*Oreiprason* | *A. tianschanicum* Rupr. | | Kyrgyzstan: 7km north-northwestward from Too-Ashuu, 04 Aug 2017, *H.J. Choi et al.* (CWNU) |
| *Reticulatobulbosa*/*Campanulata* | *A. dolichostylum* Vved. | | Kyrgyzstan: Sary-chclek, Chatkal range, 11 Aug 2016, *G.A. Lazkov* (CWNU) |
| *Reticulatobulbosa*/*Reticulatobulbosa* | *A. strictum* Schrad. | | Mongolia: Khuvsgul province, Khatgal sum, Khuvsgul Lake, 17 Jul 2015, *Sh. Baasanmunkh et al.* (CWNU) |


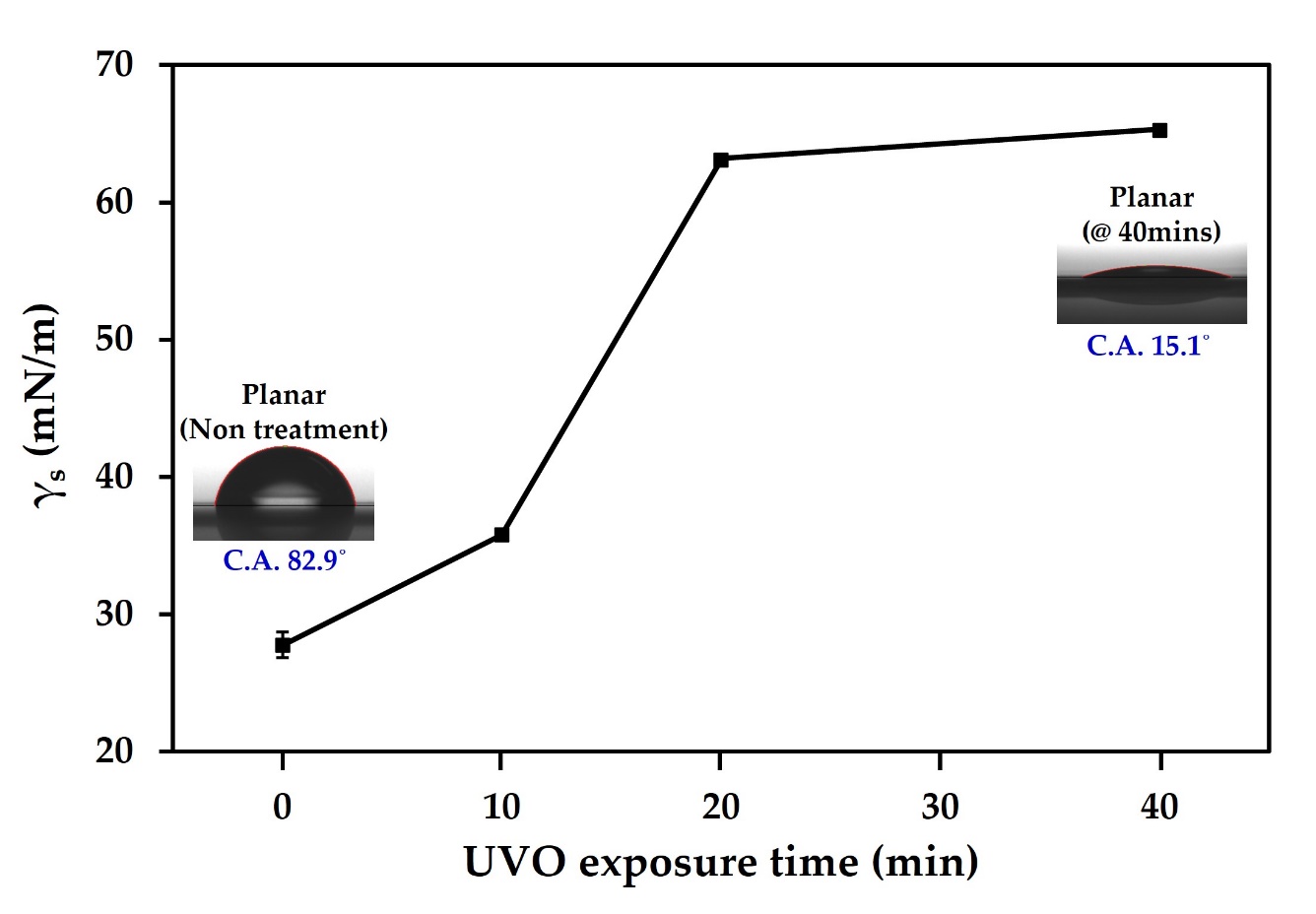


**Figure S1.** Surface energy as a function of UVO exposure time; the surface energy was 27.77 mN/m and then increased to 35.8, 63.08, and 65.23 mN/m after UVO exposure of 10, 20, and 40 min


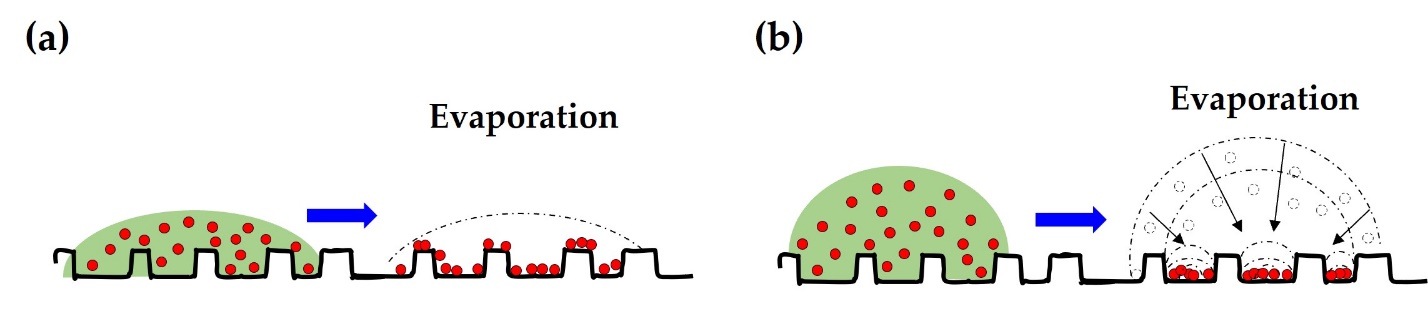


**Figure S2.** Schematics for particle residues on (a) hydrophilic and (b) hydrophobic surfaces.
